# Genomes from uncultivated prokaryotes: a comparison of metagenome-assembled and single-amplified genomes

**DOI:** 10.1186/s40168-018-0550-0

**Published:** 2018-09-28

**Authors:** Johannes Alneberg, Christofer M. G. Karlsson, Anna-Maria Divne, Claudia Bergin, Felix Homa, Markus V. Lindh, Luisa W. Hugerth, Thijs J. G. Ettema, Stefan Bertilsson, Anders F. Andersson, Jarone Pinhassi

**Affiliations:** 1 School of Engineering Sciences in Chemistry, Biotechnology and Health, Department of Gene Technology, Science for Life Laboratory, KTH Royal Institute of Technology, Stockholm, Sweden; 2 Centre for Ecology and Evolution in Microbial Model Systems, EEMiS, Linnaeus University, Kalmar, Sweden; 3 Department of Cell and Molecular Biology, SciLifeLab, Uppsala University, Uppsala, Sweden; 4 Present address: Science for Life Laboratory, Department of Molecular, Tumour and Cell Biology, Centre for Translational Microbiome Research, Karolinska Institutet, Solna, Sweden; 5 Present address: Department of Biology, Lund University, Lund, Sweden; 6 Department of Ecology and Genetics, Limnology, Science for Life Laboratory, Uppsala University, Uppsala, Sweden

**Keywords:** Single-amplified genomes, Metagenome-assembled genomes, Metagenomics, Binning, Single-cell genomics

## Abstract

**Background:**

Prokaryotes dominate the biosphere and regulate biogeochemical processes essential to all life. Yet, our knowledge about their biology is for the most part limited to the minority that has been successfully cultured. Molecular techniques now allow for obtaining genome sequences of uncultivated prokaryotic taxa, facilitating in-depth analyses that may ultimately improve our understanding of these key organisms.

**Results:**

We compared results from two culture-independent strategies for recovering bacterial genomes: single-amplified genomes and metagenome-assembled genomes. Single-amplified genomes were obtained from samples collected at an offshore station in the Baltic Sea Proper and compared to previously obtained metagenome-assembled genomes from a time series at the same station. Among 16 single-amplified genomes analyzed, seven were found to match metagenome-assembled genomes, affiliated with a diverse set of taxa. Notably, genome pairs between the two approaches were nearly identical (average 99.51% sequence identity; range 98.77–99.84%) across overlapping regions (30–80% of each genome). Within matching pairs, the single-amplified genomes were consistently smaller and less complete, whereas the genetic functional profiles were maintained. For the metagenome-assembled genomes, only on average 3.6% of the bases were estimated to be missing from the genomes due to wrongly binned contigs.

**Conclusions:**

The strong agreement between the single-amplified and metagenome-assembled genomes emphasizes that both methods generate accurate genome information from uncultivated bacteria. Importantly, this implies that the research questions and the available resources are allowed to determine the selection of genomics approach for microbiome studies.

**Electronic supplementary material:**

The online version of this article (10.1186/s40168-018-0550-0) contains supplementary material, which is available to authorized users.

## Background

The genome is a fundamental resource for understanding the physiology, ecology, and evolution of an organism. With the availability of high-throughput sequencing technologies, we are witnessing a massive increase in the number of genomes in public repositories, with nearly a doubling per year in the Genomes OnLine Database (GOLD) [1, 2]. Reference genomes are important in both medical and environmental microbiology for capturing information on metabolic properties [3], phylogeny [4], evolution and diseases [5, 6], population genetics [7], functionality and biogeochemical cycles [8], and interactions [9] and to establish links between genomes and functionality of cells in organisms [10]. In fact, obtaining good and relevant reference genomes is crucial for current advances in many, if not all, branches of biological research [11].

Prokaryotes dominate the biosphere in the context of abundance and diversity [12] and hold key roles in biogeochemical processes essential to all life [13]. However, only a small fraction of the bacterial diversity (< 1%) can be isolated and cultivated in a standardized fashion [14]. Therefore, strategies for recovering genomes from samples without the need for cultivation have emerged as important complements to traditional microbiological techniques. In the single-amplified genome (SAG) strategy, genomes of individual cells are sequenced. The first step comprises partitioning of the cells [15–17] using techniques such as fluorescent-activated cell sorting (FACS) [18, 19] or microfluidics [20]. The next step involves cell lysis and whole-genome amplification (WGA) for which three methods are most commonly used: PCR-based (e.g., degenerate oligonucleotide-primed PCR (DOP-PCR)), isothermal (e.g., multiple displacement amplification (MDA)), or hybrid methods (e.g., multiple annealing and looping-based amplification cycles (MALBAC)) [21] before applying shotgun sequencing and genome assembly [20, 22].

Genomes can also be recovered from metagenomes by assembling short shotgun reads into longer contigs which are then clustered into groups, or bins, of contigs derived from the same organism, through a process called binning. The resulting bins are quality filtered for contamination and completeness, and the approved bins are referred to as metagenome-assembled genomes (MAGs), a term proposed by Hugerth et al. [23] and later accepted by the Genomic Standards Consortium (GSC) [24]. Metagenomic binning has been used for some time [25], but a fairly recent development is to perform the binning using a combination of sequence composition and differential abundance information [26–29]. Whereas it is possible to use as few as two samples for utilizing differential abundance information, the quality of the binning results can be greatly improved by increasing the number of samples [27, 28].

Although both the SAG and the MAG approaches have proven powerful and contributed greatly to our understanding of the physiology and evolution of organisms [23, 30–35], a number of challenges are associated with each approach. SAG sequencing is demanding in terms of instrumentation and staff [36]. Starting with only one genome copy makes DNA amplification necessary but difficult, which often results in highly uneven coverage depth and some regions being completely missing from the sequencing output [21, 37]. The commonly used method for DNA amplification, multiple displacement amplification (MDA), has also been shown to cause formation of chimeric molecules, mainly through inversions [38]. Contamination is a common problem with SAG sequencing, originating either from reagent kits [39] or from free DNA in environmental samples [20]. Furthermore, cell dispersion, which might be necessary when cells are attached to particles or have formed biofilms, can be problematic and hinder genome recovery from some single cells [40]. Obtaining a large number of high-quality MAGs, on the other hand, requires extensive sequencing and ideally a large number of samples that to some degree share the same organisms in different abundances [28]. The quality of the MAGs is also highly dependent on the quality of the metagenome assembly; short contigs are not considered by most binning algorithms since their coverage and composition information contain too much noise [28, 41, 42]. Another limitation is the computational demands, which normally exceed those for SAG assembly [41]. Also, due to intraspecies genetic variation in the community, genomes recovered from metagenomic data often represent a population of closely related organisms (i.e., strains) rather than an individual organism [41].

Studies have successfully combined the SAG and MAG approaches to reach conclusions about organisms and ecosystems [43, 44]. The approaches have also been combined to methodologically improve either the quality of the single-cell assemblies [45] or the metagenome binning performance [46]. However, with the exception of a study that focused on a single phylum and that did not use abundance patterns over multiple samples for the MAG construction [43], the performance of the two approaches have to our knowledge not been thoroughly compared. The aim of this study was to do a comprehensive comparison between the SAG and MAG approaches for recovering prokaryotic genomes. We investigated SAGs and MAGs from bacterioplankton collected in the Baltic Sea Proper, where recent analyses have provided a detailed picture of the spatio-temporal distribution of microbial populations [23, 47–49] and metabolic processes [50]. Thus, this ecosystem is well suited for comparing different methodologies for investigating the genomic content and functional potential of dominant bacterial populations.

## Results

### Overview of SAGs and MAGs

In order to compare single-amplified genomes with metagenome-assembled genomes from the same environment, we generated SAGs from the Linnaeus Microbial Observatory (LMO), located 11 km off the coast of Sweden in the Baltic Sea, and compared them with MAGs generated earlier from the same station [23]. We obtained 16 SAGs of a variety of taxa including *Bacteroidetes*, *Cyanobacteria*, *Alphaproteobacteria*, and *Gammaproteobacteria* (Additional file 1: Table S1). These were compared to 83 MAGs from 30 phylogenetically distinct Baltic Sea clusters (BACLs) [23] (Additional file 2: Figure S1; Additional file 1: Table S1). The SAGs ranged in size from 0.14 to 2.15 Mbp and MAGs from 0.59 to 2.98 Mbp (Additional file 1: Table S1). The number of contigs in SAGs ranged from 80 to 712 with a maximum length of 107,141 bp, while the number of contigs in MAGs ranged from 60 to 951 with the longest being 181,472 bp (Additional file 1: Table S1).

Using Mash [51] to cluster the 99 genomes from both approaches, seven of the 16 SAGs were placed together with 24 of the MAGs into six clusters (i.e., each of these SAGs matching 1–14 MAGs and each of these MAGs matching 1–2 SAGs; Table 1 and Additional file 2: Figure S1). This was in agreement with the clustering of MAGs in the analysis of Hugerth et al. [23]. These clusters belonged to a diverse set of bacterial taxa, representing the SAR86 and SAR92 clades (*Gammaproteobacteria*), *Flavobacteriaceae* (2 taxa) and *Cryomorphaceae* (*Bacteroidetes*) and *Rhodobacteraceae* (*Alphaproteobacteria*) (Table 1). The following comparisons between SAGs and MAGs are based on the genomes in these clusters.

**Table 1:** Overview of the matching SAGs and MAGs sorted by Baltic Sea cluster (BACL) number

	Nucleotide identity in % (standard deviation)	Size (in bp)	% completeness	% redundancy	% MAG aligned	% SAG aligned	% SAG reads mapping			
							MAG contigs	≥ 1kb contigs outside MAG	< 1 kb contigs	Not mapping to metagenome
BACL1: *Gammaproteobacteria*; SAR86										
BS0038H10		547073	30.22	0.72						
120507-bin14	99.36 (1.71)	1482147	94.24	2.16	29.10	84.20	72.07	0.01	18	9.92
120619-bin26	99.62 (1.21)	1539140	92.81	0.72	28.06	82.49	73.96	0.25	17.57	8.22
120813-bin36	99.56 (0.81)	1264266	92.09	1.44	31.19	79.38	76.61	0.33	7.68	15.38
120820-bin45	99.48 (1.15)	1455539	92.81	0.72	29.10	82.30	74.03	0.01	15.3	10.66
120823-bin87	99.55 (1.04)	1451966	93.53	2.16	29.53	85.14	78.2	0.04	11.46	10.3
120828-bin5	99.58 (0.77)	1029940	85.61	0.72	32.47	68.11	71.16	5.53	9.48	13.83
120920-bin57	99.57 (1.26)	1450272	86.33	4.32	27.45	76.21	68.46	0.15	23.47	7.92
120924-bin88	99.61 (0.97)	1314100	91.37	0.72	30.30	79.59	72.71	0.01	15.61	11.67
121001-bin56	99.57 (1.19)	1509054	87.05	4.32	28.33	82.35	70.28	0.04	18.97	10.71
121004-bin11	99.68 (0.46)	1030921	78.42	0.00	32.44	68.15	66.78	10.84	11.58	10.8
121015-bin70	99.49 (1.44)	1495089	92.81	0.00	29.02	86.08	80.22	0.01	10	9.76
121022-bin58	99.58 (0.93)	1435343	93.53	0.72	29.37	84.33	78.28	0.01	10.77	10.94
121105-bin34	99.61 (0.73)	1306513	94.24	4.32	30.45	79.11	74.96	0.01	13.64	11.39
121128-bin56	99.54 (1.19)	1469346	94.24	4.32	29.37	85.59	76.36	0.01	13.33	10.3
BACL7: *Bacteroidetes*; *Cryomorphaceae*; *Owenweeksia*										
A11		1656754	68.35	7.91						
120322-bin74	99.84 (0.21)	1743356	97.84	0.00	75.05	83.52	87.6	0.42	2.25	9.73
120910-bin2	99.82 (0.31)	1746953	97.12	0.72	75.05	83.53	87.4	1.07	1.7	9.83
121220-bin83	99.82 (0.24)	1723929	95.68	0.00	75.13	82.22	85.79	3.22	2.62	8.37
BACL10: *Alphaproteobacteria*; *Rhodobacter*										
BS0038D5		1732939	39.57	0.72						
120419-bin15	99.18 (1.16)	2834045	96.40	2.88	42.11	68.10	53.62	2.53	22.67	21.18
120910-bin24	99.21 (1.02)	2763624	95.68	1.44	42.24	68.31	55.24	0.58	21.91	22.27
121220-bin24	99.07 (0.95)	2112289	84.89	1.44	46.50	58.12	45.37	1.79	24.68	28.16
BACL16: *Gammaproteobacteria*; SAR92										
BS0038E9		1153566	41.73	1.44						
120322-bin99	99.45 (0.74)	1997685	92.09	1.44	42.50	74.24	70.42	2.3	17.57	9.71
120619-bin48	99.20 (1.51)	2527476	99.28	0.72	40.23	89.12	86.03	2.77	1.81	9.39
BACL21: *Bacteroidetes*; *Flavobacteriaceae*										
BS0038D11		1637880	74.82	2.16						
121220-bin10	99.75 (0.38)	1915951	97.84	0.72	75.00	88.18	84.21	2.15	6.66	6.98
BS0038D2		1023978	37.41	2.16						
121220-bin10	99.74 (0.46)	1915951	97.84	0.72	45.59	85.40	92.43	1.37	4.36	1.85
BACL22: *Bacteroidetes*; *Flavobacteriaceae*; *Polaribacter*										
BS0038A11		1334036	33.81	2.88						
120619-bin32	98.77 (2.13)	2408986	97.12	3.60	39.15	72.59	66.22	0.17	7.98	25.63
Average	99.51 (0.96)	SAG: 1298032	SAG: 46.56	SAG: 2.57	40.59	79.05	73.94	1.42	12.44	12.20
		MAG: 1716955	MAG: 92.83	MAG: 92.83						

The seasonal dynamics of the clusters at the LMO station were determined in the original MAG study by metagenome samples covering a single year (2012) [23]. By comparing the 16S rRNA gene sequences from the genome clusters to 16S rRNA gene data from an amplicon-based high-temporal-resolution study from the same station from the previous year (2011) [49], we observed five matches with a sequence identity of 100%. In these cases, the seasonal dynamics of the genome clusters and OTUs was similar between the years, with representatives abundant in spring and late autumn (2012) (BACL21, *Flavobacteriaceae,* OTU:000004 and BACL7, *Owenweeksia*, OTU:000021); spring and early summer (BACL16, SAR92 clade, OTU:000043); spring, summer, and autumn (BACL10, *Rhodobacteraceae*, OTU:000011); and all year round (BACL1, SAR86 clade, OTU:000013) [23, 49] (Additional file 3: Figure S2). The contigs representing the genomes of BACL22 lacked the 16S rRNA gene sequence and were not included in the seasonality analysis.

### Alignment and gene content

To verify the clustering and to achieve more detailed statistics, each SAG-MAG pair was aligned using MUMmer (Table 1). Across the genome regions showing homology between SAGs and MAGs, the within-cluster nucleotide sequence identity averaged 99.51%, with the lowest sequence identity value recorded for BACL22 (98.77%; Table 1). A larger fraction of the SAGs’ bases (average 78.9%) aligned compared to the MAGs’ (average 40.5%), in agreement with these SAGs being consistently smaller than the corresponding MAGs, 0.5–1.7 Mbp and 1.0–2.8 Mbp, respectively (Table 1)[23].

**Fig. 1:**
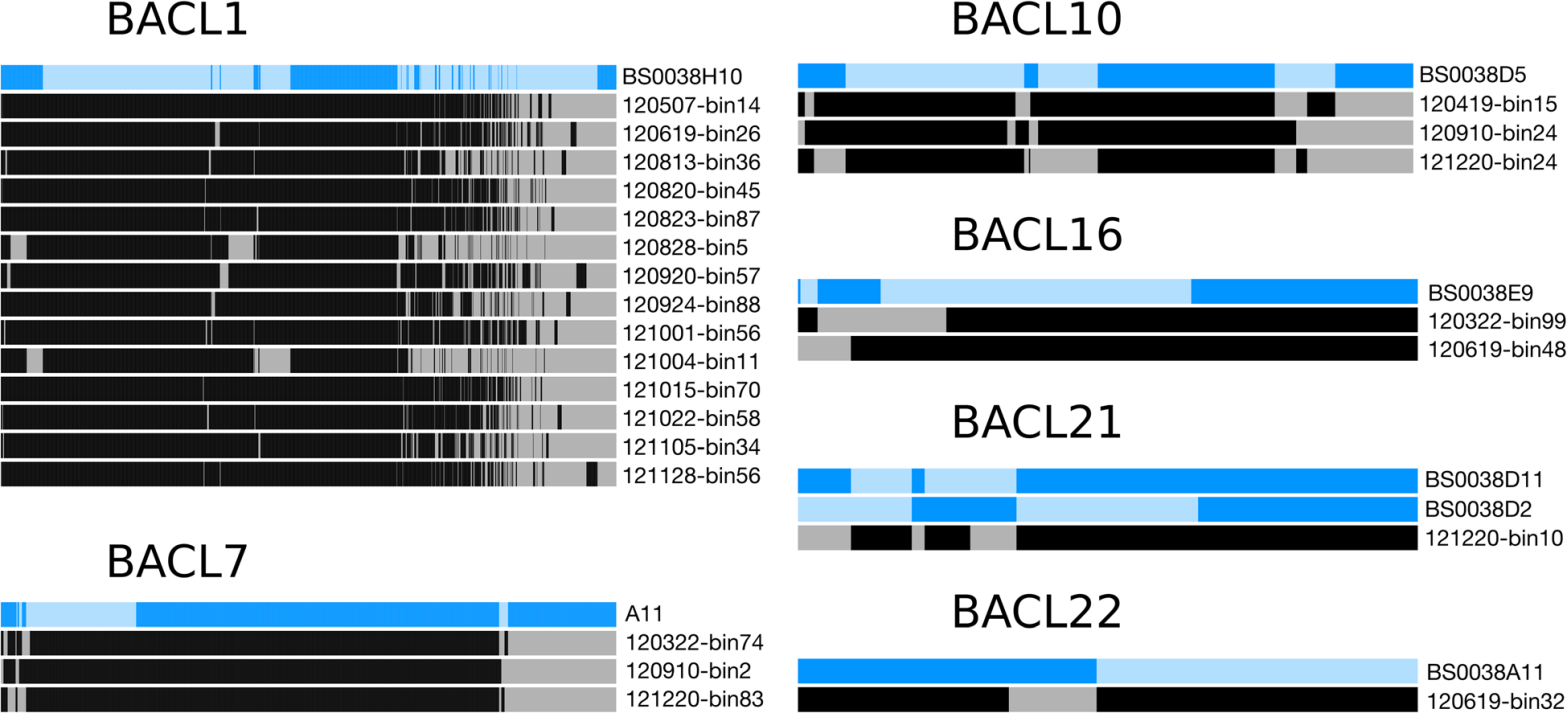
Gene homolog presence per genome cluster. Presence of gene homologs for each genome cluster by graphs produced by Anvi’o. Each horizontal bar represents one genome, where blue bars are single-amplified genomes and black and grey bars are metagenome-assembled genomes. Each vertical bar corresponds to one gene homolog where a dark vertical bar indicates presence of the gene homologs and a lighter vertical bar indicates absence. The gene homologs are aligned between genomes within each genome cluster. The numbers assigned to the genome clusters corresponds to the original MAG BACLs used in [23]

To further compare the SAGs and MAGs, the Anvi’o pangenomic workflow [52] was run on each cluster (Fig. 1, Additional file 4: Table S2). This analysis showed that the completeness of the SAG genomes (average 46.6%) was lower than that of the MAG genomes (average 92.6%) (Table 1), as estimated by Anvi’o (by presence of 139 bacterial single-copy genes [SCGs]). Redundancy in gene content (measured as SCGs present more than once) showed no systematic difference between SAGs and MAGs (Additional file 4: Table S2); it was highest in SAG A11 and in four MAGs of BACL1 (with 7.9% and 4.3%, respectively). For details on contamination of SAGs, see the Results section “SAG quality evaluation” below.

There was a substantial range in gene content overlaps in different clusters (Fig. 1). For example, most MAGs in BACL1 contained a large set of genes (~ 35% of genomes) missing in the corresponding SAG (BS0038H10), whereas the SAG in this cluster contained few genes (~ 5% of genomes) not present in the MAGs. In contrast, in BACL7, similar portions of the genes (~ 20% of genomes) were unique to the SAG or the MAGs. The case of BACL21 is particularly interesting since it contained two SAGs (the only cluster with more than one SAG) that differed substantially in size (1.0 Mb and 1.6 Mb; Table 1). The two SAGs together covered nearly the entire gene content of the corresponding MAG (Fig. 1).

For the genomes that were placed in the six clusters, 16S rRNA genes were found in four out of seven SAGs (57%) and 19 out of 24 MAGs (79%), where the latter proportion is notably high. In comparison, analysis of 16S rRNA genes in all genomes showed that 11 out of 16 SAGs (69%) and 38 out of 83 MAGs (46%) contained 16S rRNA gene sequences. It is worth noting that the higher proportion of SAGs containing a 16S rRNA gene sequence in the complete dataset could reflect that the initial selection of SAGs for sequencing was mainly based on them containing a PCR-amplifiable 16S rRNA gene sequence. A lower proportion for MAGs could also be due to known issues with metagenome assembly and binning of sequences from 16S rRNA genes [53].

### Analysis of functional gene data

**Fig. 2:**
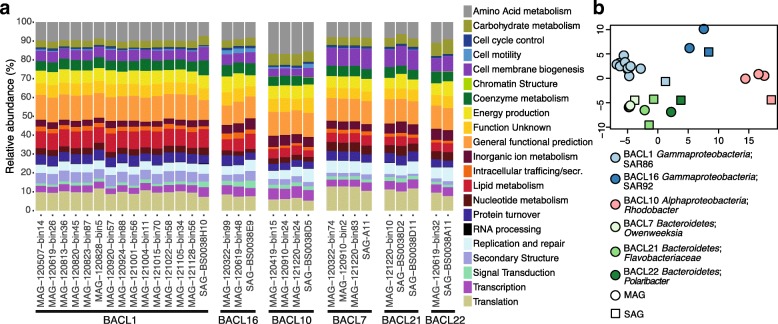
Distribution of functional categories in SAGs and MAGs. **a** Distribution of broad COG categories in the different genome clusters for MAGs and SAGs. The *X*-axis shows genomes grouped and ordered according to genome clusters. The *Y*-axis shows the percentage of genes in COG categories in each genome. **b** Non-metric multidimensional scaling (NMDS) plot based on counts of COG categories in the SAGs and MAGs

Despite the differences in genome sizes, the distribution of broad functional gene categories, as defined by Clusters of Orthologous Groups (COGs), was largely consistent within SAG and MAG clusters (Fig. 2a). Statistical analysis of the COG category distributions showed that the genomes clustered according to BACL (ANOSIM *R* = 0.96; *P* = 0.0001) but not significantly so according to genome type (i.e., SAG vs. MAG; ANOSIM *R* = 0.21; *P* = 0.06; Fig. 2b). The distribution of COG categories also appeared to differ taxonomically (Fig. 2b). For instance, the COG category “Amino acid metabolism and transport” was more abundant in the cluster BACL10 (*Rhodobacter*) compared to other clusters. The *Flavobacteria* (BACL7, 21, and 22) showed elevated proportions of the functions “Cell wall/membrane/envelope-biogenesis” and “Translation.” “Lipid metabolism” was more frequent in the *Gammaproteobacteria* clusters (BACL1 and 16) compared to other clusters (Fig. 2a).

### Quantification of metagenome binning and assembly errors

**Fig. 3:**
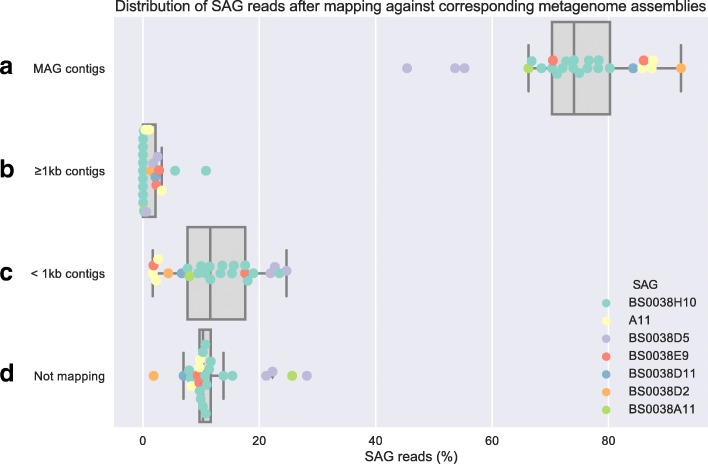
Distribution of SAG reads mapped against metagenome assemblies. Boxplot of the distribution of SAG reads mapped against the corresponding metagenome assemblies where each individual data point is jittered on top of each box. All reads for each SAG was mapped against the assembly associated with each matching MAG and thus positioned in exactly one out of these four categories. Only contigs longer than 1 kb were included in the binning, which is the reason to use it as a divider here

Since the SAGs contained genome regions not present in the MAGs (on average 78.9% of SAG genomes aligned with the corresponding MAG genomes), we investigated potential reasons for these regions to be missing in the MAGs. Accordingly, we determined the distribution of SAG sequencing reads mapping to different categories of metagenome contigs. This quantification showed that a median of 74.0% of the SAG reads mapped to the contigs in their corresponding MAG (Fig. 3a). Other metagenome contigs which were included in the binning due to their lengths (> 1 kb), but that had hence ended up in other bins, recruited far fewer reads (median 0.33%) (Fig. 3b). These contigs were likely misplaced in the binning procedure and can be used to calculate an estimate for the false negative error of the binning. This was calculated as the number of nucleotide bases in these potentially misplaced contigs covered by SAG reads divided by the number of nucleotide bases covered by SAG reads in all contigs that were subject to binning—this value averaged 3.6% (Additional file 5: Table S3). The remaining SAG reads were either mapping to small contigs (1 kb), not included in the binning because they were too short (< 1 kb) (median 11.6% of reads), or not mapping to metagenome contigs at all (median 10.3% of reads) (Fig. 3c, d) and were hence rather reflecting insufficient metagenomic assembly or contaminations in the SAGs.

### SAG quality evaluation

A potential explanation for the SAG specific content could be contaminating DNA in the SAGs [20, 39]. In order to address this, we analyzed patterns of nucleotide composition and metagenome coverage for SAG contigs. A clear difference in tetranucleotide pattern was observed between the SAG contigs that were aligning and those that were not aligning with MAG contigs. The set of SAG contigs which did not align (< 5% of bases) contained many outliers in the tetranucleotide PCA (Additional file 6: Figure S3, Additional file 7: Figure S4, Additional file 8: Figure S5, Additional file 9: Figure S6, Additional file 10: Figure S7, Additional file 11: Figure S8 and Additional file 12: Figure S9). This could potentially be due to the use of tetranucleotide patterns in the construction of MAGs and that these regions are falsely missing in the MAGs due to their atypical sequence composition. However, investigating the mapping of metagenome reads against the SAG contigs showed that the SAG contigs that were not aligning to MAGs and that displayed atypical tetranucleotide patterns also as a rule had significantly lower coverage in the metagenome (Additional file 6: Figure S3, Additional file 7: Figure S4, Additional file 8: Figure S5, Additional file 9: Figure S6, Additional file 10: Figure S7, Additional file 11: Figure S8 and Additional file 12: Figure S9). This substantially strengthens the hypothesis that these SAG contigs are due to contamination.

During MDA, chimeric sequences, in particular inversions, can be generated [38]. Using the SAG reads mapping against the metagenome, such chimeric reads were identified. Using conservative criteria, on average, 1.72% of the reads were identified as chimeric (Additional file 13: Table S4). The chimeric nature of these reads could potentially affect the mapping to the metagenome (Fig. 3), particularly by inflating the number of reads which did not map to any metagenome contig. However, the distribution of these chimeric reads among the categories of Fig. 3 did not differ from all reads (Additional file 13: Table S4). Hence, chimeric SAG reads did not bias the distribution of SAG reads mapping against the metagenome.

The chimeric reads could potentially negatively impact the SAG assembly, because the chimeras could be propagated to the contigs formed in the assembly. Since a large majority of the chimeric reads (on average 96.9%, compared to 8.6% of other reads; Additional file 14: Table S5) aligned with more than 20 bases soft-clipped against the SAG contigs, this does not seem to generally be the case. The chimeric reads could also result in a fragmented assembly by introducing alternative, erroneous, paths in the assembly graph, leading to truncated contigs. If this would be the case, one would expect chimeric reads to be overrepresented among reads overlapping the ends of contigs. While chimeric reads were more often aligned over an edge of a contig compared to other reads, these reads only corresponded to on average 6.3% of the chimeric reads. Thus, chimeric reads do not seem to have had a substantial impact on the SAG assembly.

From visual inspection of genome alignments, we discovered some MAG contigs aligning to multiple SAG contigs. This was caused by erroneously duplicated contig sequences within the SAGs, where the highest amount was found within the A11 SAG assembly. However, this issue was resolved when using the most recent version of the assembly software (Spades version 3.10.1 instead of version 3.5), tested on the A11 SAG (data not shown).

## Discussion

In this study, we compared the genome output from two state-of-the-art approaches for obtaining prokaryotic genomes representing abundant populations in the natural environment without cultivation. From a collection of SAGs and MAGs, we found an overlap in six clusters, representing a broad taxonomic range including *Gammaproteobacteria*, *Bacteroidetes*, and *Alphaproteobacteria* that were nearly identical between the two groups (average 99.51% sequence identity), verifying previous results with high average nucleotide identity between SAGs and MAGs [43]. It is interesting to note that average nucleotide identity (ANI) is an important measure of the genomic level of relatedness in the taxonomy of prokaryotes [54]. Moreover, Konstantinidis and Roselló-Móra [55] state that “In general, two organisms sharing ANI values above 94–96% may be considered as members of the same genospecies” citing the articles [56] and [57], and Varghese et al. [58] found intra-species identities to range between 96.5 and 100% ANI. Thus, it appears that the matching SAGs and MAGs in our study are highly likely to represent the same genomic populations—yet, this remains to be explored in detail in future phylogenomic analyses. Due to seasonal recurrence of bacterial populations in the waters studied here [23, 49], a very high nucleotide identity (> 98.7% in overlapping regions) could be achieved despite samples used for SAG sequencing and MAG construction were collected 1 year apart. From the relative abundance of matching data on specific bacterial populations (OTUs), we conclude that both approaches provide genomic information on abundant taxa in the natural environment.

There are, however, differences between the two methods. When conducting sequencing of single-amplified genomes, one of the benefits is that the cells can be screened and the researcher can select particular cells to sequence, perhaps targeting a specific taxon or function. Furthermore, if one has only very few samples, producing SAGs may be preferable since the efficiency of the MAG approach improves with the number of samples [28]. Similarly, the MAG approach has critical difficulties assembling closely related strains [59] and the presence of multiple strains also inhibits accurate binning [28]. Moreover, closely related strains that display a wide variation in genetic content may obtain different abundance patterns, since temporal dynamics may differ between core- and strain-specific parts of the genomes. SAGs also supply superior information on which nucleotide variants that co-occur within a genome (haplotypes), whereas for metagenomics, this information is limited to the read length, although computational approaches for haplotype reconstruction are emerging [60]. Nevertheless, metagenome-assembled genomes do recover a higher percentage of the genome compared to SAGs. Also, since reads from many individuals of each population are being sampled, population genomic analysis can be performed using the metagenome data [61–63], and additional information about the whole microbial community is obtained from the metagenome dataset, which is achieved with a more standard set of equipment compared to that needed for single-cell sequencing. Multiple samples are often beneficial for ecological investigations, making such projects suitable for MAG construction. Nevertheless, the fact that the genomes matched abundant OTUs with representatives from different taxonomic groups shows that both the SAG and the MAG approaches have a broad generality when applied to environmental samples.

### Size of SAGs compared to MAGs

The SAGs in this study were consistently smaller than the corresponding MAGs. This could be caused by either incomplete SAG assemblies or by metagenome contigs erroneously placed in MAGs by the binning algorithm. Looking closer at the case where two SAGs aligned to the same single MAG (i.e., BACL21), there was evidence that the smaller of the two SAGs (BS0038D2) was incomplete, i.e., it lacked a large fraction of genes that were shared by the second SAG and the MAG (Fig. 1). Our results therefore support the first explanation, which has been previously observed [39, 64, 65]. Combining the sections included in the SAGs would also cover a higher proportion of the MAG than any of the two SAGs did individually (Fig. 1). Furthermore, MAGs showed a low level of redundancy (i.e., measured as duplicated SCGs) which would likely have been higher if MAGs contained a high degree of erroneously binned contigs. Finally, matching SAGs are also less complete than MAGs as estimated by presence of SCGs.

The cause for incomplete SAGs could be either uneven or incomplete amplification of parts of the, typically, single-genome copy [21]. The average sequencing depth was, however, one order of magnitude higher for the SAGs than the MAGs (Additional file 1: Table S1), and in most cases, the sequencing reads used were longer. The formation of chimeric reads is an additional problem potentially affecting the assembly quality of SAGs. Our analysis shows, however, that while chimeric reads are present, they are in most cases not aligning over the edges of SAG contigs (Additional file 14: Table S5). It therefore seems likely that the major causes for incomplete SAGs are other problems related to whole-genome amplification. Attempts to improve this method are ongoing [66, 67], but alternatively, multiple SAGs from the same population can be sequenced for better coverage [31, 37]. Even though the SAGs were smaller than MAGs, the analysis of COG categories within each matching SAG and MAG demonstrated that the two approaches capture the broad functional categories in a similar manner (Fig. 2). This essentially indicates that a majority of functional genes in different categories are fairly evenly distributed across the genomes.

### Unique SAG sequences—metagenome assembly problem or contamination?

With the caveats that the whole-genome amplification of single cells generates uneven depth of coverage for different parts of the genome [21], mapping SAG reads against the metagenomes allowed us to investigate how well the MAGs and the remaining metagenomes accounted for all SAG sequences (Fig. 3). SAG reads mapping to contigs included in the corresponding MAG accounted for the largest fraction for all pairs of MAGs and SAGs, confirming the completeness of the MAGs (Fig. 3a). In the MAG assembly, only contigs longer than 1 kb were used as input to the binning, because short contigs are difficult to cluster correctly [28]. Therefore, reads mapping to contigs which were longer than 1 kb, and thus subject to binning, but not included in the corresponding MAG (Fig. 3b), likely indicated wrongly binned contigs or possibly indicated sequence variation between strains of the same population. A high rate of false negative binning errors would necessitate a high percentage of reads in this category. However, this was not observed (Fig. 3b). Instead, the estimated false negative rate of the binning was low—on average only 3.6% measured as number of genomic bases.

In contrast, a significant portion of the SAG reads were placed in either of the two remaining categories: reads mapping to metagenome contigs shorter than 1 kb (Fig. 3c) or reads not mapping to any metagenome contig (Fig. 3d). This could potentially be due to that metagenome assembly failed to assemble true MAG sequences past the 1-kb cutoff used for binning. Improvements of metagenome assembly strategies have recently been made [68, 69], possibly reducing the influence of this issue. Alternatively, these sequences could correspond to SAG contamination. While not easily quantified, our analysis showed clear presence of contaminating sequences within the SAGs (Additional file 6: Figure S3, Additional file 7: Figure S4, Additional file 8: Figure S5, Additional file 9: Figure S6, Additional file 10: Figure S7, Additional file 11: Figure S8 and Additional file 12: Figure S9). Contaminating sequences could either be introduced during the handling of samples in the lab [39] or be present in the environmental samples as free DNA [20]. An additional possibility is that some regions here identified as contamination of SAGs are instead true SAG sequences which are unique to the SAG genome in comparison to the MAG. Genome regions recently acquired through horizontal gene transfer are likely to have a different sequence composition [70] which is also one of the criteria to identify SAG contamination.

### No significant core genome enrichment in MAGs

A potential problem with binning using coverage variations over multiple samples is that strain-specific genes can have different abundance profiles than the core genome if multiple strains of the same species are present in the samples [28]. Therefore strain-specific and core genes are at risk of being placed into different bins, and the use of single-copy core genes as an estimate of completeness would result in an overly optimistic measure for the core genome bin. If any of the MAGs would be artificially core-genome-enriched in the binning procedure, we would expect a large fraction of the SAG reads, in particular those corresponding to the non-core genome, to map to the long contigs that were not in the MAGs. This was however not the case, as only a very small fraction was detected (Fig. 3b). These findings indicate that core genome enrichment in the construction of MAGs is a smaller problem than previously thought. However, the severity of this problem is likely dependent on the structure of the pangenome of the organism.

## Conclusion

Individual MAGs in this study were found to be larger and more complete than corresponding SAGs, although there is reason to believe that analysis of multiple SAGs from the same group of organisms could result in equal or higher completeness if jointly assembled. The false negative rate in the binning process was generally low. Single-cell technology offers the possibility of genome recovery from a single sample whereas the reconstruction of MAGs often requires multiple samples. This, on the other hand, provides ecological information based on the MAG abundance variations across samples. The strong agreement between the SAG and MAG methodologies emphasizes that both are accurate and that the choice of approach should depend on the research questions and on available resources.

## Methods

### Generation of MAGs

The MAGs used in the current study were obtained as previously described in Hugerth et al. [23]. Briefly, bacterial community DNA for MAG construction was obtained from surface water (2 m) collected in the Baltic Sea on 37 time points between March and December 2012 at the Linnaeus Microbial Observatory (LMO) located ~ 11 km offshore Kårehamn, Sweden (56°55′.51.24″ N 17°3′38.52″ E). Library preparation of the bacterial community DNA was performed with the Rubicon ThruPlex kit (Rubicon Genomics, Ann Arbor, MI, USA) according to the instructions of the manufacturer, and finished libraries were sequenced on a HiSeq 2500 (Illumina Inc., San Diego, CA, USA) with paired-end reads of 2 × 100 bp at SciLifeLab/NGI (Solna, Sweden). On average, 31.9 million paired-end reads per sample were generated.

Quality-controlled reads were assembled separately for each sample using a combination of Ray 2.1 (Ray Meta) [71] and 454 Life Science’s software Newbler (v.29; Roche, Basel, Switzerland). Bowtie2 [72] was used to map all quality-controlled reads for each sample against the contigs. Contigs from each sample were then binned using CONCOCT [28], an algorithm that clusters contigs into genomes across multiple samples, dependent on sample coverage and sequence composition using Gaussian mixture models. Bins were evaluated with a set of 36 single-copy genes presented in [28] and approved if they contained at least 30 unique SCGs with a maximum of 2 in more than a single copy. Bins meeting these criteria were considered MAGs. It should be noted that metagenome assembly and metagenome binning softwares continuously evolve, which could potentially influence MAG construction. However, the CONCOCT algorithm has not changed since we applied it on these data, and CONCOCT is regarded a highly successful software for metagenome binning [59, 73]. Two MAGs from different samples could correspond to the same organism, and therefore, the 83 MAGs were clustered using MUMmer [74] into 30 Baltic Sea clusters (BACL). Functional analysis of each BACL was made with the PROKKA pipeline (v.1.7) [75] and extended with annotation for COG categories [76]. Taxonomic assignment for each MAG was firstly done with Phylosift [77] and then complemented with complete or partial 16S rRNA genes identified in the MAGs with webMGA [78].

### SAG sampling and single-cell sorting

Samples for SAGs from the Baltic Sea were collected on 13 May 2013 at the Linnaeus Microbial Observatory and cryopreserved in 1× TE, 5% glycerol (final concentration) before arriving to the Microbial Single Cell Genomics facility, SciLifeLab, Uppsala University. Prior to sorting, the cryopreserved samples were thawed and diluted, before being stained with 1× (final concentration) SYBR Green I (Life Technologies, CA, USA) for approximately 30 min. The sorting was performed with a MoFlo Astrios EQ (Beckman Coulter, USA) cell sorter using a 488-nm laser for excitation, 70-μm nozzle, sheath pressure of 60 psi, and 1.3% sterile filtered NaCl as sheath fluid. Individual cells were deposited into 96-well plates (Bio-Rad, CA, USA) containing 1 μL of 1× TE using a CyClone™ robotic arm and the most stringent single-cell sort settings (single mode, 0.5 drop envelope). The sorter was triggered on forward scatter at a threshold of 0.08%, and sort regions were set on SYBR Green I fluorescence detected at 513 nm using a 40-nm bandpass filter.

### Whole-genome amplification using MDA with phi29

Deposited cells were lysed and neutralized followed by whole-genome amplification using Phi29 and MDA as described by [18]. In short, the cells were incubated in an alkaline solution at RT for 5 min. Lysis reactions were neutralized by adding 1 μL neutralization buffer (Qiagen, Germany). MDA was performed using the RepliPHI™ Phi29 Reagent set (0.1 μg/μL, RH04210, Epicenter, WI, USA) at 30 °C for 16 h in 15 μL reaction volumes with a final concentration of 1× reaction buffer, 0.4 mM dNTPs, 10 μM DTT, 5% DMSO, 50 μM hexamers with 3′-phosphorothioate modifications (IDT Integrated DNA Technologies, IA, USA), 40 U Phi 29 enzyme, 0.5 μM SYTO13® (Life Technologies, CA, USA), and water. All reagents except SYTO13 were UV decontaminated at 2 × 0.5 J in a Biolinker. The whole-genome amplification was monitored in real time by detection of SYTO13 fluorescence every 15 min for 16 h using a Chromo4 real-time PCR instrument (Bio-Rad, CA, USA). The single amplified genome DNA was stored at − 20 °C until further PCR screening, library preparation, and Illumina sequencing.

### Screening of SAGs

Positive SAGs, defined by an early amplification curve well separated from negative controls as well as a positive PCR product targeting the 16S rRNA gene, were diluted 20-fold and screened using primer pair Bact_341 F: 5′-CCTACGGGNGGCWGCAG-3′ and Bact_805 R: 5′- GACTACHVGGGTATCTAATCC-3′ [47]. The reactions were performed in 20 μL reaction volume with 2 U of Taq DNA Polymerase recombinant (Thermo Fisher Scientific, MA, USA), 1× reaction buffer, 0.2 mM dNTPs, 2 mM MgCl_2_, and 0.25 μM of each primer. Following a 3-min denaturation at 95 °C, targets were amplified for 35 cycles of 95 °C for 30 s, 50 °C for 30 s, 72 °C for 60 s, and a final 10-min extension at 72 °C. PCR products were detected by an approximate 450-bp fragment on a 1.5% agarose gel. The products were purified using the NucleoSpin Gel and PCR clean-up purification kit (Macherey-Nagel, Germany), quantified using the Quant-iT ™ PicoGreen® dsDNA assay kit (Invitrogen, MA, USA) in a FLUOstar® Omega microplate reader (BMG Labtech, Germany) and submitted for identification by Sanger sequencing at the Uppsala Genome Center.

### Illumina MiSeq sequencing

Altogether, 15 SAGs were selected for genome sequencing. Twelve of these generated a 16S rRNA sequence identified by Sanger sequencing and were selected to cover a broad range of phylogenetic groups. Three additional SAGs did not generate any 16S rRNA amplicons with the indicated primers but were nevertheless selected to include also lineages not targeted by bacterial primers.

The DNA content of the SAGs was quantified with the Quant-iT ™ PicoGreen® dsDNA assay kit and subsequently diluted to a concentration of 0.2 ng/μL as recommended for the Nextera XT Library Preparation kit (Illumina, CA, USA). Procedures were according to instructions from the manufacturer except that normalization was performed using the Kapa qPCR quantification method instead of bead normalization. In short, the Nextera XT uses an enzymatic step for fragmentation of DNA which enables small quantities of input DNA. The protocol involves a PCR amplification step where indexes and additional required nucleotide sequences are incorporated. After PCR cleanup, the library for each SAG was quantified and handed in for individual quality control at the SciLifeLab SNP&SEQ facility. The quality of the libraries was evaluated using the TapeStation from Agilent Technologies with the D1000 ScreenTape. The sequencing libraries were quantified by qPCR using the library quantification kit for Illumina (KAPA Biosystems, MA, USA) on a StepOnePlus instrument (Applied Biosystems, CA, USA) and pooled in equal amounts prior to cluster generation and sequencing on a single MiSeq run with V3 chemistry and 2 × 300 bp mode.

One additional SAG (A11) from the same sample but from another sorted plate was purified using the NucleoSpin Tissue purification kit (Macherey-Nagel, Germany) and handed in directly to the SNPseq sequencing facility for preparation using the TruSeq Nano DNA library kit (Illumina, CA, USA) and thereafter sequenced in another MiSeq V3 2 × 300 bp run.

### Data analysis of sequenced libraries

The global quality of raw and trimmed reads was checked using Fastqc 0.11 [79], and low-quality data was removed together with adapters using Cutadapt 1.7 [80], requiring a minimal length of 75 nucleotides and using a quality of 30 as the threshold. The trimmed reads were assembled using the default values for single cell (*--sc*) with SPAdes 3.5 [81] and the parameter *careful*, which, according to the documentation, reduces the number of mismatches and short indels in contigs. The quality of each of the assemblies was assessed using the software QUAST 2.3 [82].

### Comparative genomics analyses

Mash version 1.0.1 [51] with 100,000 15-mers for each SAG and MAG was used to calculate pairwise distances between all genomes. Single-linkage clustering was then performed using Scipy [83] and visualized using matplotlib [84] (Additional file 2: Figure S1). Clustering cutoff for each BACL was set at 0.1 (90% estimated similarity), and in each cluster containing a combination of MAGs and SAGs, they were pairwise aligned using the dnadiff tool from the Mummer suite version 3.23 [74]. Since Mash only gives an estimation of the nucleotide distance, we also subjected two additional clusters just over the 10% dissimilarity limit (BACL24 and BACL30) for alignment with MUMmer. Out of these, BACL30 resulted in the best alignment at 96.5% identity and alignment rate of the SAG at 53.7%. However, none of these two clusters were included in the comparison. The numbers assigned to the clusters correspond to the original MAG BACLs used in [23]. None of the SAGs or MAGs was closely related to complete genomes available through the newly developed Genome Taxonomy Database (http://gtdb.ecogenomic.org/). We only found some matches to non-SAG/non-MAG genomes for BACL16. The matches of the BACL16 120322 MAG to the genomes of the two bacterial strains MOLA455 and HTCC2207 were less than 2% and 4% of the aligned bases, respectively (determined using MUMmer/dnadiff); the sequence identity was < 83% across the aligned regions.

Following the same procedure as [23], the SAGs were gene annotated using the PROKKA pipeline [75] and complemented with all significant (*e* value < 0.00001) COG annotations using rpsblast from BLAST+ version 2.2.28+ [85]. Non-metric multidimensional scaling (NMDS) and ANOSIM analysis was based on counts of COG categories in the genomes, running the ANOSIM with 99,999 permutations. The pairwise genome distances for these analyses were calculated using Poisson dissimilarity [86] with the PoiClaClu package, and NMDS and ANOSIM were conducted with the Vegan package, in *R* (www.r-project.org). Using the Anvi’o (Docker image with version 2.1.0) pangenomic workflow [52, 87] separately for each genome cluster, gene homologs were identified and visualized and estimates of completeness and redundancy were obtained using the MCL algorithm [88], prodigal [89], hmmer [90], and 139 bacterial single-copy genes (SCGs) defined by [91]. The summary statistics produced by Anvi’o are available in Additional file 4: Table S2.

SAG reads corrected during the assembly process [81] that mapped to the SAG genome itself (minimum 99.55%) were mapped using Bowtie2 (version 2.2.6 with the --local argument) [72] against the assembled metagenome samples from which the MAGs were obtained. The resulting BAM-files were sorted using Samtools version 1.3 [92], duplicates were removed with Picard version 1.118, and the number of mapped reads per contig was counted (Fig. 3). Metagenomic contigs were divided into three groups: contigs included in the correct MAG, long (≥ 1 kb) contigs included in the binning but not belonging to the correct MAG, and short (< 1 kb) contigs not included in the binning. Additionally, there were those reads that did not map to the metagenome assembly at all. The counts were summarized and visualized using Pandas [93] and Seaborn [94].

Duplicated elements in the genomes were identified with BLASTN version 2.2.28+ [85] as alignments longer than 0.1 kb between contigs longer than 1 kb and with 100% nucleotide identity. Reassembly of A11 was done using the corrected reads from existing assembly as input to Spades version 3.10.1 run in single-cell mode.

### Prevalence of 16S rRNA gene sequences in SAGs and MAGs and seasonal occurrence

Twelve out of the 16 single-amplified genomes had 16S rRNA genes identified through Sanger sequencing as described above. However, four SAGs (A11, BS0038A02, BS0038A08, and BS0038A11) seemed to lack 16S rRNA gene sequence data and were therefore investigated with Barrnap (version 0.8) [95]. Barrnap identified the 16S rRNA gene in SAG A11 and this sequence was taxonomically investigated using the SINA/SILVA database [96]. Barrnap was also applied to all SAGs and MAGs to compare the presence of 16S rRNA genes in the genomes.

To obtain a taxonomic annotation for the three remaining SAGs without 16S rRNA genes (BS0038A02, BS0038A08, and BS0038A11), we investigated their good quality contigs with a minimum length of 1 kb and kmer coverage (provided by Spades) of at least 11. Prodigal 2.6.1 [89] was then used to predict coding regions in the selected contigs and predicted proteins were aligned against NCBI nucleotide and NCBI non-redundant database using BLAST (standalone BLAST + package version 2.2.30) [85].

To investigate the presence in the Baltic Sea of the 13 SAGs having a 16S rRNA gene, we individually blasted the sequences to a 16S rRNA gene amplicon dataset from a field study at the LMO station [49] using online BLASTN [97]. The seasonal dynamics were then explored by comparing the matching SAG/MAG clusters from 2012 (i.e., BACLs from Hugerth et al. 2015 [23]) to the corresponding OTU in 2011 (i.e., Lindh et al. 2015 [49]).

### Analysis of contamination of SAGs and chimeric reads

The presence of contamination within SAGs was visually estimated through a tetranucleotide nucleotide composition PCA. The PCA was performed on all contigs from each individual SAG, but for visualization, the contigs were separated into two sets. One set contained contigs which aligned with less than 5% of their lengths to their corresponding MAG and the other set contained all other contigs (which did align to the corresponding MAG). When more than one MAG were in the same cluster, the union of all aligning SAG bases was used. To make the contamination detection less dependent of the MAGs, the contigs were also colored according to the number of metagenome reads mapping to them. The average metagenome coverage was estimated by assuming a length of 100 bases for each metagenome read. For a clearer visualization, the high coverage values were adjusted so that the maximum value was only three times the median value. The density plots were constructed using Pandas [93].

To identify chimeric SAG reads that contain inversions, the SAG read mappings against the metagenomes were investigated. Reads were first flagged as potentially chimeric if they mapped with at least 20 soft-clipped bases (as marked with S in the SAM-file cigar string) against any of the metagenome samples where matching MAGs had been obtained. Furthermore, the remaining matching region of the read was required to correspond to at least half of the read length and contain no more than two mismatches. This rather strict requirement was enforced to minimize the risk that the mapping was not to the intended organism. Finally, the list of potentially chimeric reads from all matching metagenome samples was combined, deduplicated, and filtered to remove reads which mapped in a non-chimeric fashion in any sample. We defined a non-chimeric mapping to contain alignment of at least 95% of the read length and to contain less than five mismatches within this region.

The effect of chimeric reads was evaluated on both SAG assembly and on statistics for mapping SAG reads against metagenome. For all mapping files, the distribution of the chimeric reads was evaluated based on whether they were soft clipped and whether their alignment was overlapping a contig edge. A soft-clipped alignment was defined as containing at least 20 clipped bases. Furthermore, for mapping files of SAG reads against the metagenome samples, categories were defined based on the metagenome contigs in analogy to Fig. 3. A metagenomic contig was either shorter than 1 kb, longer than 1 kb but not contained within the focal MAG, or part of the MAG in question. These categories were used to investigate the distribution of mapping SAG reads based on the metagenome contig they mapped against.

## Additional files

Additional file 1: **Table S1.** Assembly statistics and taxonomy for all MAGs and SAGs. For MAGs, “Coverage within sample” indicates that coverage was calculated based on the sample from where it was assembled. (XLSX 14 kb)

Additional file 2: **Figure S1.** Hierarchical single-linkage clustering of SAGs and MAGs based on distances generated by MASH. Genome names starting with “BACL” indicate MAGs and the number following indicates the Baltic Sea cluster. Leaves joined by nodes within a distance of 0.10 are grouped by color of their leftmost branches. (PDF 71 kb)

Additional file 3: **Figure S2.** Abundances over the years 2011 and 2012 for OTUs matching clusters of SAGs and MAGs. Redrawn from references Hugerth et al. and Lindh et al. [23, 49]. (PDF 188 kb)

Additional file 4: **Table S2.** Summary statistics as given by Anvi’o for all MAGs and SAGs found by both approaches. (XLSX 23 kb)

Additional file 5: **Table S3.** Distribution of metagenome bases covered by SAG reads mapped against the corresponding metagenome assemblies. “Estimated False Negative Rate in Binning (%)” was calculated by dividing the number of “Bases covered within long (≥ 1 kb) non-MAG contigs” with the number of “Nucleotide bases covered within MAG contigs.” (XLSX 10 kb)

Additional file 6: **Figure S3.** Tetranucleotide frequency plots of SAG BS0038H10 in BACL1. Nucleotide composition PCAs (a,b) and metagenome coverage estimate density plots (c,d) for contigs separated on alignment rate (< 5% of bases: a,c; ≥5% of bases: b,d) against the corresponding MAG. The color of the circles in panels a and b corresponds to the average metagenome coverage and the size of the circles corresponds to the contig sizes. Metagenome average coverage depth was estimated by assuming all mapping reads were 100 bases long. Furthermore, for clarity, the maximum value for the average coverage depth has been set to three times the median. (PDF 144 kb)

Additional file 7: **Figure S4.** Tetranucleotide frequency plots of SAG A11 in BACL7. Other figure legend information same as in Additional file 6: **Figure S3.** (PDF 192 kb)

Additional file 8: **Figure S5.** Tetranucleotide frequency plots of SAG BS0038D5 in BACL10. Other figure legend information same as in Additional file 6: **Figure S3.** (PDF 236 kb)

Additional file 9: **Figure S6.** Tetranucleotide frequency plots of SAG BS0038E9 in BACL16. Other figure legend information same as in Additional file 6: **Figure S3.** (PDF 100 kb)

Additional file 10: **Figure S7.** Tetranucleotide frequency plots of SAG BS0038D2 in BACL21. Other figure legend information same as in Additional file 6: **Figure S3.** (PDF 158 kb)

Additional file 11: **Figure S8.** Tetranucleotide frequency plots of SAG BS0038D11 in BACL21. Other figure legend information same as in Additional file 6: **Figure S3.** (PDF 87 kb)

Additional file 12: **Figure S9.** Tetranucleotide frequency plots of SAG BS0038A11 in BACL22. Other figure legend information same as in Additional file 6: **Figure S3.** (PDF 141 kb)

Additional file 13: **Table S4.** Statistics for chimeric SAG reads mapping against metagenome contigs. (XLSX 14 kb)

Additional file 14: **Table S5.** Statistics for chimeric SAG reads and other SAG reads mapping against SAG contigs. (XLSX 11 kb)

## Abbreviations

BACL: Baltic Sea cluster
COG: Clusters of Orthologous Groups
LMO: Linnaeus Microbial Observatory
MAG: Metagenome-assembled genome
Mbp: Million base pairs
OTU: Operational taxonomic unit
SAG: Single-amplified genome
