# Supplementary figures and images for: Genomes from uncultivated prokaryotes: a comparison of metagenome-assembled and single-amplified genomes
Source: Microbiome. 2018 Sep 28;6:173. doi: 10.1186/s40168-018-0550-0 (PMC6162917; doi:10.1186/s40168-018-0550-0)

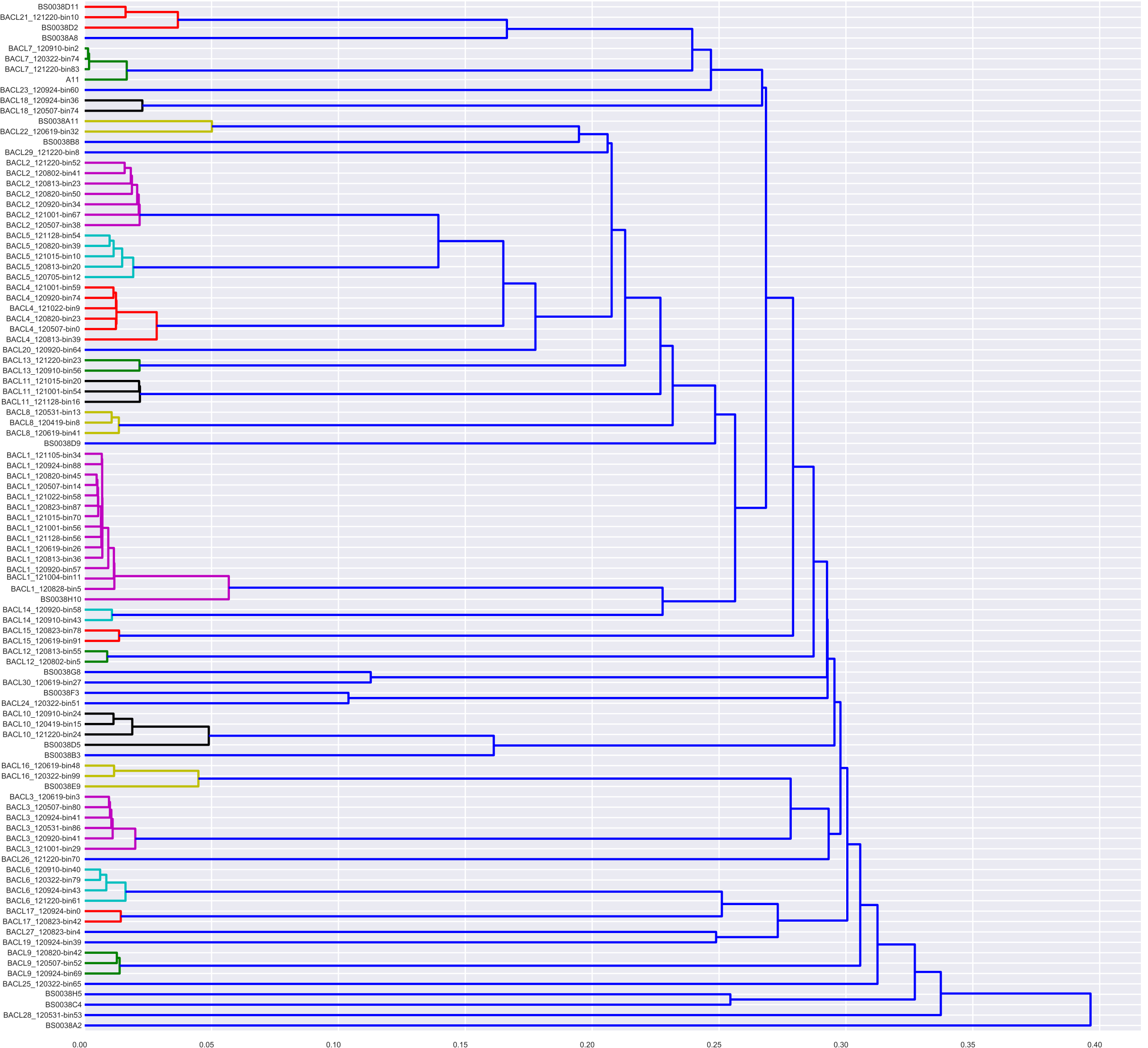

Supplement: Supplementary file 2 — Figure S1. Hierarchical single-linkage clustering of SAGs and MAGs based on distances generated by MASH. Genome names starting with “BACL” indicate MAGs and the number following indicates the Baltic Sea cluster. Leaves joined by nodes within a distance of 0.10 are grouped by color of their leftmost branches. (PDF 71 kb) [file 40168_2018_550_MOESM2_ESM.pdf]

# BACL1 - BS0038H10

SAG Contigs with < 5% aligned to MAG

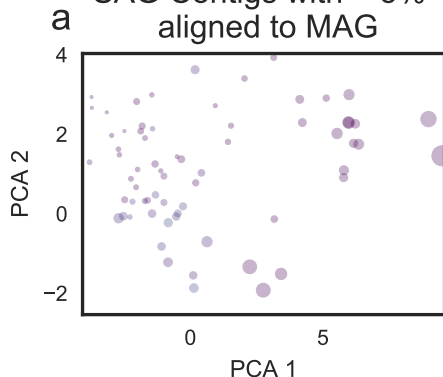

SAG Contigs with > 5% aligned to MAG

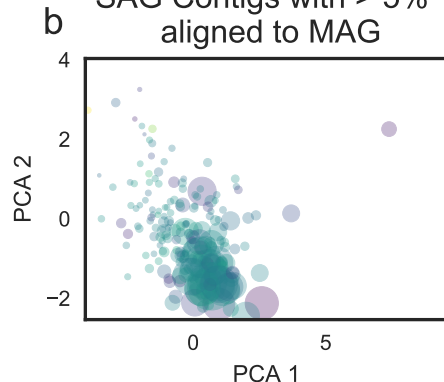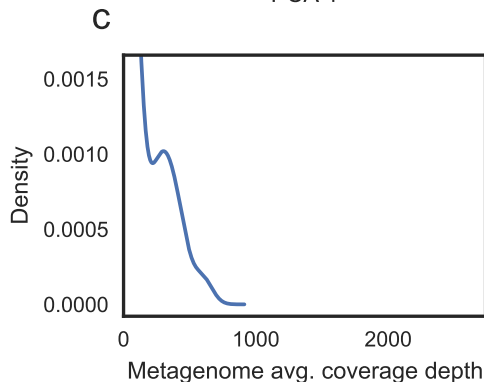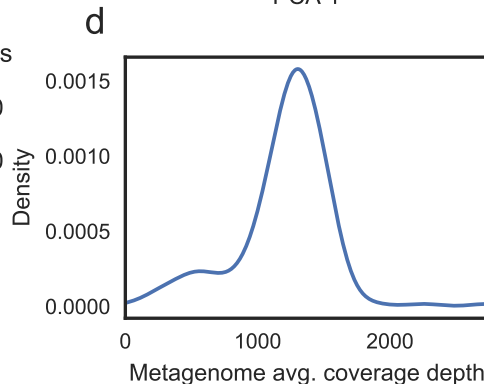

Supplement: Supplementary file 6 — Figure S3. Tetranucleotide frequency plots of SAG BS0038H10 in BACL1. Nucleotide composition PCAs (a,b) and metagenome coverage estimate density plots (c,d) for contigs separated on alignment rate (< 5% of bases: a,c; ≥5% of bases: b,d) against the corresponding MAG. The color of the circles in panels a and b corresponds to the average metagenome coverage and the size of the circles corresponds to the contig sizes. Metagenome average coverage depth was estimated by assuming all mapping reads were 100 bases long. Furthermore, for clarity, the maximum value for the average coverage depth has been set to three times the median. (PDF 144 kb) [file 40168_2018_550_MOESM6_ESM.pdf]

# BACL7 - A11

SAG Contigs with < 5% aligned to MAG

a

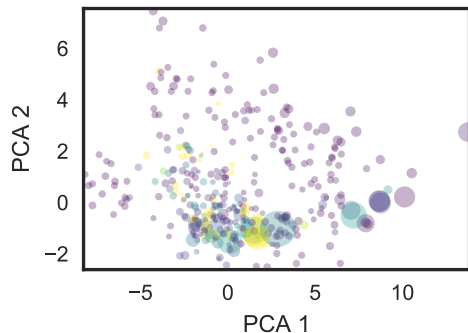

SAG Contigs with > 5% aligned to MAG

b

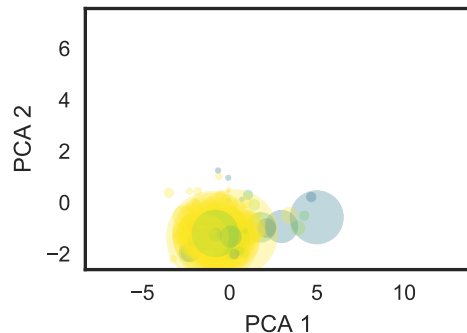

c

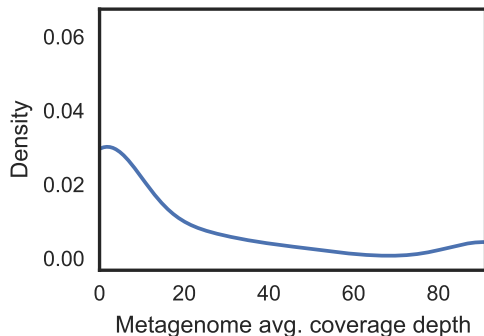

Contig lengths

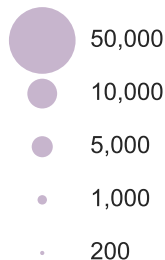

d

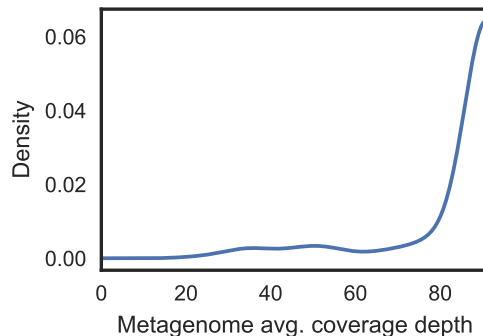

Supplement: Supplementary file 7 — Figure S4. Tetranucleotide frequency plots of SAG A11 in BACL7. Other figure legend information same as in Additional file 6: Figure S3. (PDF 192 kb) [file 40168_2018_550_MOESM7_ESM.pdf]

# BACL10 - BS0038D5

SAG Contigs with < 5%  
aligned to MAG

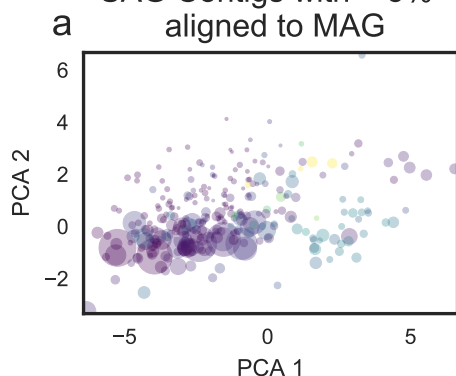

SAG Contigs with > 5%  
aligned to MAG

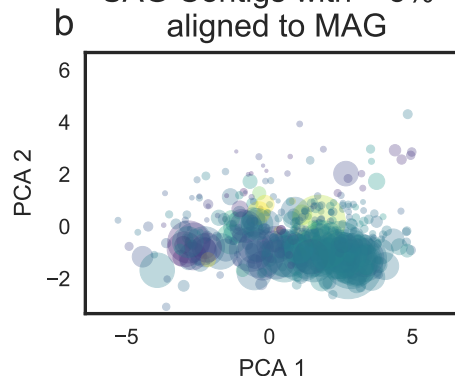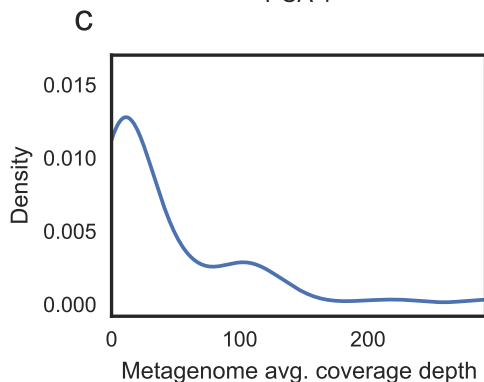

Contig lengths

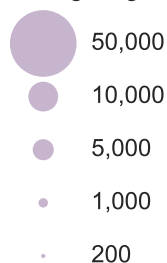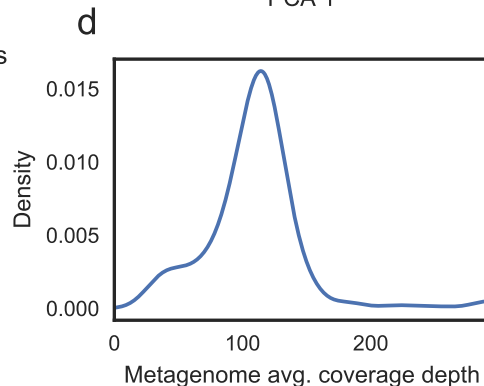

Supplement: Supplementary file 8 — Figure S5. Tetranucleotide frequency plots of SAG BS0038D5 in BACL10. Other figure legend information same as in Additional file 6: Figure S3. (PDF 236 kb) [file 40168_2018_550_MOESM8_ESM.pdf]

SAG Contigs with < 5%  
aligned to MAG

a

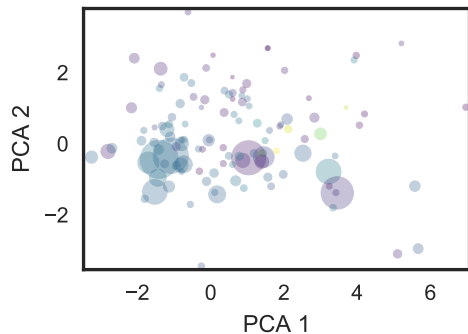SAG Contigs with > 5%  
aligned to MAG

b

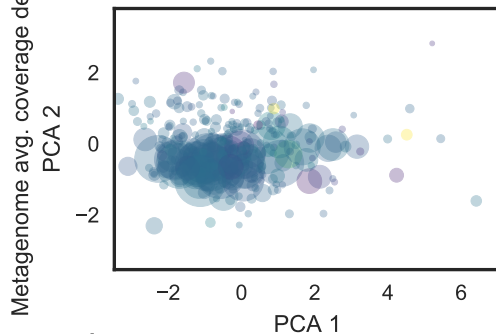

c

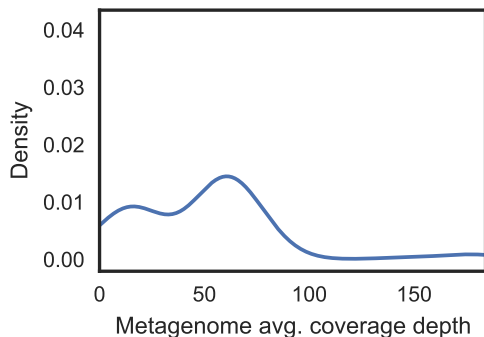

Contig lengths

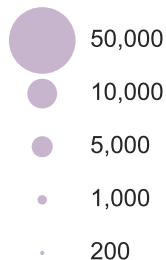

d

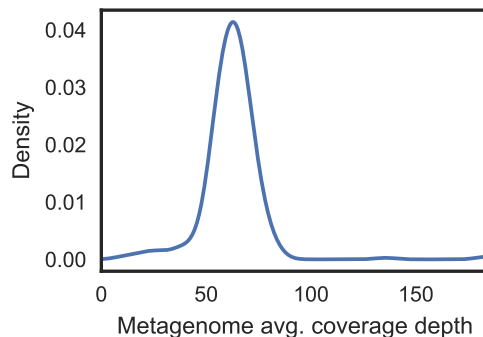

Supplement: Supplementary file 9 — Figure S6. Tetranucleotide frequency plots of SAG BS0038E9 in BACL16. Other figure legend information same as in Additional file 6: Figure S3. (PDF 100 kb) [file 40168_2018_550_MOESM9_ESM.pdf]

# BACL21 - BS0038D2

SAG Contigs with < 5%  
aligned to MAG

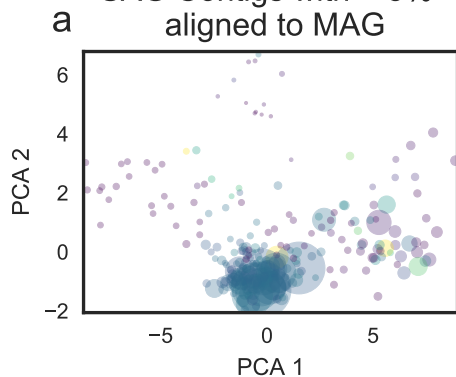

SAG Contigs with > 5%  
aligned to MAG

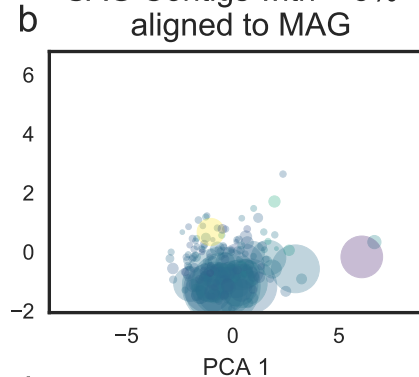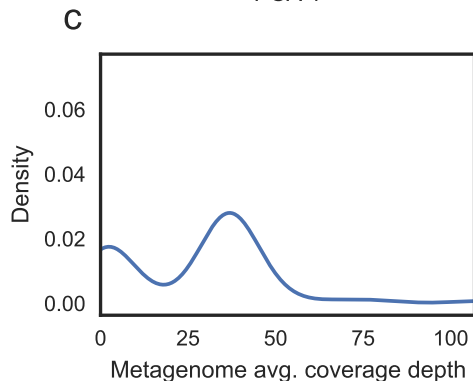

Contig lengths

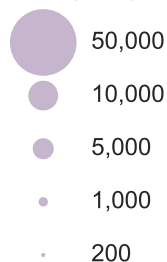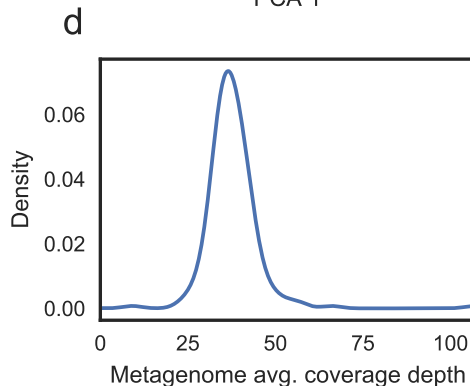

Supplement: Supplementary file 10 — Figure S7. Tetranucleotide frequency plots of SAG BS0038D2 in BACL21. Other figure legend information same as in Additional file 6: Figure S3. (PDF 158 kb) [file 40168_2018_550_MOESM10_ESM.pdf]

SAG Contigs with < 5%  
aligned to MAG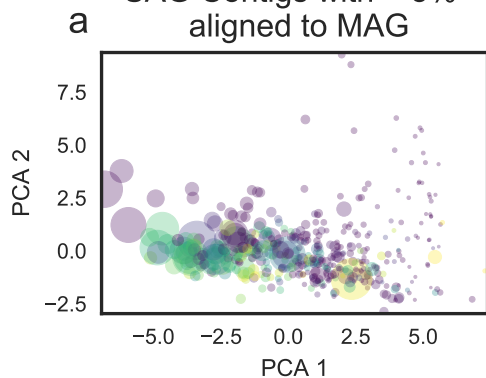SAG Contigs with > 5%  
aligned to MAG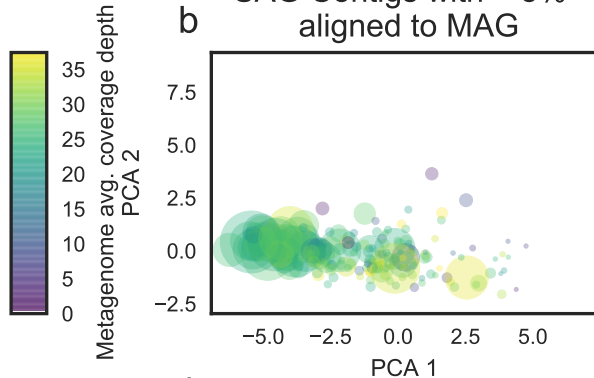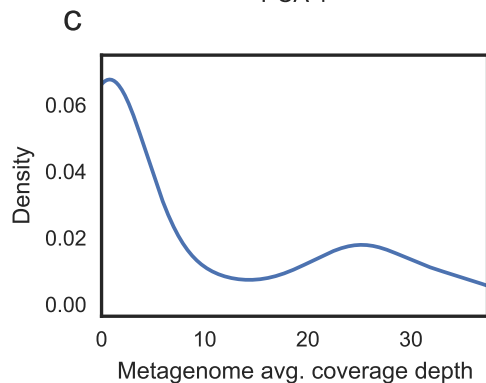

Contig lengths

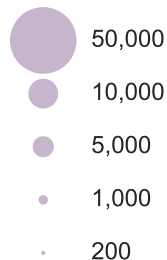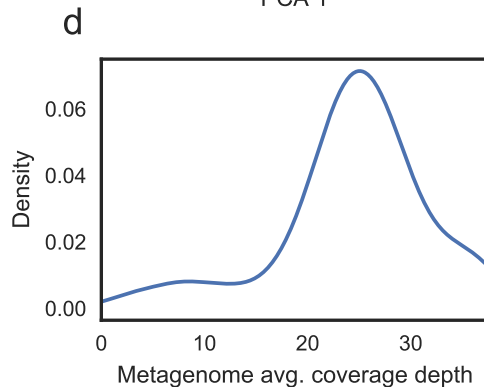

Supplement: Supplementary file 12 — Figure S9. Tetranucleotide frequency plots of SAG BS0038A11 in BACL22. Other figure legend information same as in Additional file 6: Figure S3. (PDF 141 kb) [file 40168_2018_550_MOESM12_ESM.pdf]
