# Supplementary material for: Genomes from uncultivated prokaryotes: a comparison of metagenome-assembled and single-amplified genomes
Source: Microbiome. 2018 Sep 28;6:173. doi: 10.1186/s40168-018-0550-0 (PMC6162917; doi:10.1186/s40168-018-0550-0)
Supplement: Supplementary file 3 — Figure S2. Abundances over the years 2011 and 2012 for OTUs matching clusters of SAGs and MAGs. Redrawn from references Hugerth et al. and Lindh et al. [23, 49]. (PDF 188 kb) [file 40168_2018_550_MOESM3_ESM.pdf]

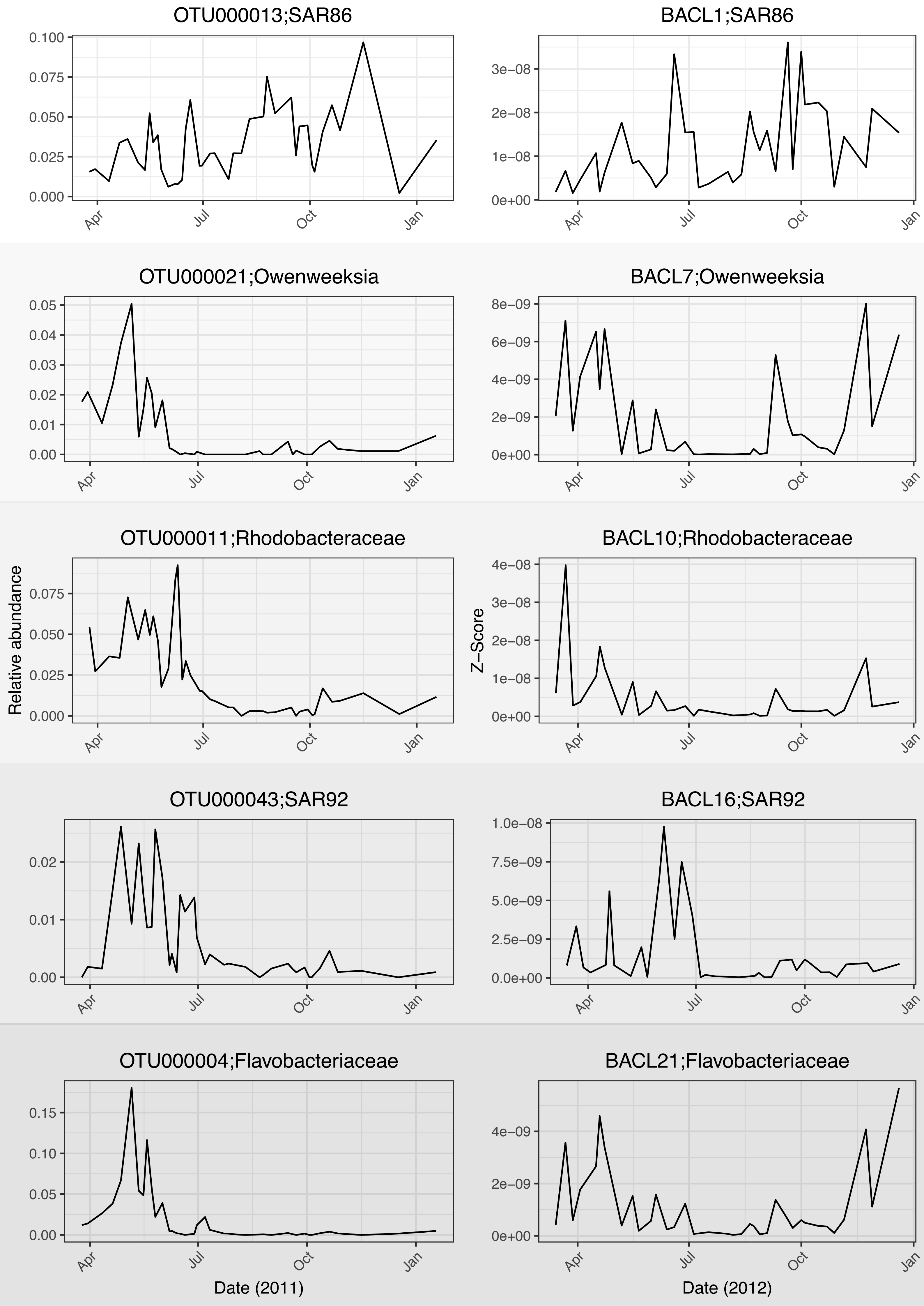

OTU000013;SAR86
BACL1;SAR86
OTU000021;Owenweeksia
BACL7;Owenweeksia
OTU000011;Rhodobacteraceae
BACL10;Rhodobacteraceae
OTU000043;SAR92
BACL16;SAR92
OTU000004;Flavobacteriaceae
BACL21;Flavobacteriaceae
Relative abundance
Z−Score
Apr
Jul
Oct
Jan
Date (2011)
Date (2012)
