# Supplementary material for: Genomes from uncultivated prokaryotes: a comparison of metagenome-assembled and single-amplified genomes
Source: Microbiome. 2018 Sep 28;6:173. doi: 10.1186/s40168-018-0550-0 (PMC6162917; doi:10.1186/s40168-018-0550-0)
Supplement: Supplementary file 11 — Figure S8. Tetranucleotide frequency plots of SAG BS0038D11 in BACL21. Other figure legend information same as in Additional file 6: Figure S3. (PDF 87 kb) [file 40168_2018_550_MOESM11_ESM.pdf]

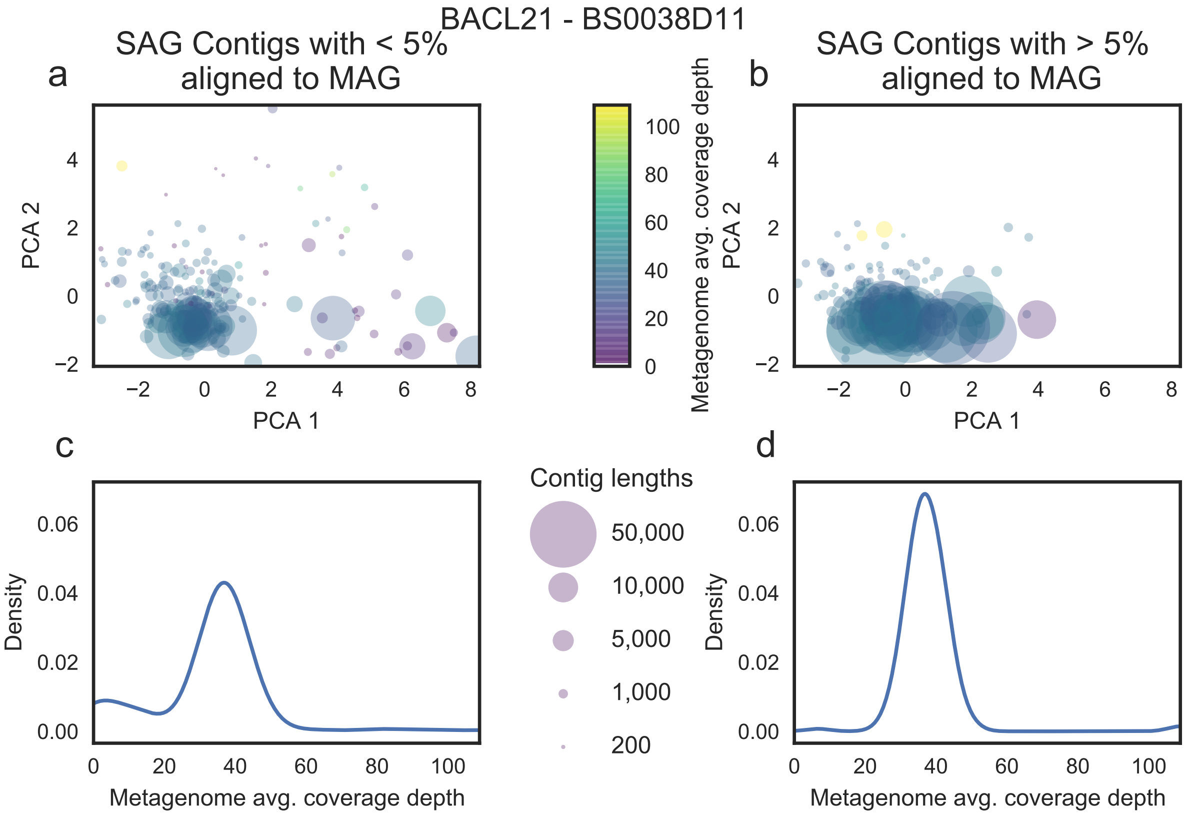

BACL21 - BS0038D11
a
SAG Contigs with < 5% aligned to MAG
b
SAG Contigs with > 5% aligned to MAG
PCA 2
PCA 1
Metagenome avg. coverage depth
c
d
Contig lengths
50,000
10,000
5,000
1,000
200
Density
Metagenome avg. coverage depth
